# Acute palliative and supportive care units provide unique and tailored care: A case report

**DOI:** 10.1017/S1478951525101211

**Published:** 2026-01-20

**Authors:** Shaylee Dave, Kayley M. Ancy, Ahsan Azhar, Eduardo Bruera

**Affiliations:** Department of Palliative, Rehabilitation and Integrative Medicine, The University of Texas MD Anderson Cancer Center, Houston, TX, USA

**Keywords:** Acute palliative and supportive care units, transitions in care, palliative care, interdisciplinary team, supportive care

## Abstract

**Objective:**

Acute Palliative and Supportive Care Units (APSCUs) provide unique and tailored care to patients with advanced chronic illness. In an APSCU, patients receive intensive palliative care while remaining in an acute care hospital setting. This allows for physical and psychological suffering to be aggressively treated by a dedicated interdisciplinary team. In the case of our patient, the APSCU changed the trajectory of his expected outcome of in-hospital death to a successful discharge home.

**Methods:**

We report the case of a patient with advanced colon cancer who suffered cardiac arrest in the emergency department and was expected to die soon after transition to comfort-oriented care.

**Results:**

After terminal extubation, our patient continued to have agitated delirium and was transferred to our APSCU for aggressive symptom control. He stabilized and progressively became more responsive, prompting a change in his plan of care with a goal of discharge home with hospice. The APSCU’s interdisciplinary team was able to adapt to the patient’s unexpected clinical improvement and provide him and his family with the medical, psychosocial, and spiritual expertise to prepare him for a successful discharge home, where he ultimately died 6 weeks later.

**Significance of results:**

The case report demonstrates that an APSCU, with its skilled interdisciplinary team in the acute care hospital, is an ideal setting to provide patient-centered care for seriously ill patients and their families.

## Introduction

Patients with advanced illnesses such as cancer experience multiple complex symptoms, particularly near the end of life. These challenges may include not only physical but also profound psychological, social, and spiritual components, such as issues with complex advanced care planning and prognostication, loss of autonomy, meaning in life, and spiritual or existential crises (Zimmermann et al. 2014). These ailing patients, along with their caregivers and family members, require intense interdisciplinary care for favorable outcomes.

The purpose of this case report is to highlight the impact of acute palliative and supportive care units (APSCUs) equipped with skilled interdisciplinary staff who work together toward improvement in patient-reported outcomes in an acute care setting designed specifically for the needs of seriously ill patients and their families (Casarett et al. 2011; Chai and Meier 2011; Zimmermann et al. 2014).

## Case description

A man in his 40s with a history of metastatic colon cancer that had progressed through multiple lines of therapy presented to the emergency center for a malfunctioning port-a-cath. He had developed bowel obstruction requiring a venting gastrostomy tube and was receiving intermittent intravenous fluids to maintain his volume status and quality of life. During the emergency center evaluation, he developed acute respiratory distress requiring intubation and mechanical ventilation, followed quickly by cardiac arrest. Return of spontaneous circulation was achieved after a few minutes, and he was transferred to the intensive care unit, where our palliative and supportive care team was consulted to assist with care planning conversations.

Given his advanced disease and lack of remaining treatment options, his family desired a transition to comfort-oriented care, and he underwent palliative extubation. He remained agitated in the following days, requiring high doses of continuous opioids and benzodiazepines. By hospital day 8, he was stable for transition to the APSCU, at which time his morphine equivalent daily dose (MEDD) was 2400 mg.

Upon transfer to the APSCU, his family was under the impression that he would die quickly. We initially felt similarly as a team; however, he became progressively more alert over the next hours to days. As we began decreasing his neuroleptics and opioids, his thinking became clear, he expressed thirst and hunger, and he was able to enjoy quality time with his family. Daily meetings with our interdisciplinary team (IDT) allowed him and his family to process his unexpected clinical improvement since transfer to the APSCU. We facilitated a slow shift in expectations: from the anticipated end of life in the hospital to the possibility of a successful transition home.

Our patient also had a significant amount to process emotionally – he found himself in an unexpected limbo. He continued to require frequent, sometimes escalating intravenous opioid doses for breakthrough pain, though he experienced improvement in his functional status. To help manage his pain, integrative measures were also used, including oncologic massage and acupuncture, with some improvement. We felt he was suffering spiritually and emotionally in this process, with the possibility of chemical coping further complicating his transition off parenteral medications. Consistent daily meetings with our chaplain and counselor allowed his emotions, worries, and fears to be heard and validated.

**Figure 1.**
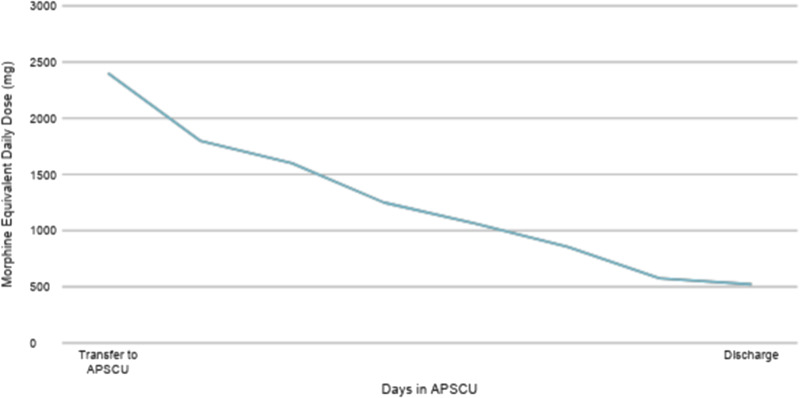
The patient’s morphine equivalent daily dose from the time of transfer to the acute palliative and supportive care unit (2400mg) to the time of discharge (522mg).

As he was slowly transitioned from continuous intravenous to oral opioids, he benefited from ongoing access to acute care hospital services. His dislodged drainage percutaneous gastrostomy tube was replaced, and he received physical and occupational therapy. Given his unexpected clinical improvement, our patient and his family met with his primary oncologist to reaffirm his lack of appropriate treatment options. These services provided the support and closure the patient and his family needed for a safe transition. On hospital day 25, he was ultimately discharged home with hospice care and lived another 6 weeks. His MEDD at the time of discharge was 522 mg (Figure 1).

## Discussion

APSCUs address the complex needs of patients with advanced chronic illness, bridging a gap in care. In an APSCU, patients receive concurrent intensive palliative care while remaining in an acute care hospital setting. Both physical and psychological suffering are aggressively treated by a dedicated IDT. In the case of our patient, the APSCU changed the trajectory of his expected outcome of in-hospital death to a successful discharge home, alive. Only 40% of National Cancer Institute–designated cancer centers and 7% of other cancer centers reported having APSCUs (Hui et al. 2023). The importance of APSCUs in providing patient-centered care should not be minimized and universal implementation should be considered.

The presence of an IDT on daily rounds allows the APSCU to provide personalized patient care and re-evaluation regarding the care plan when needed. The physician, nurse, counselor, chaplain, pharmacist, social worker, and case manager each contribute their unique expertise while collaborating in a cohesive manner to support the patient’s goals of care through thorough assessments, coordinated communication, and multidimensional interventions (Hui et al. 2018). Highly effective palliative care teams have a shared vision and adapt easily to change (Ragsdale et al. 2024). In the case of our patient, the IDT allowed frequent self-reflection regarding our approach to the patient and addressed the cognitive bias of presumed in-hospital death that we all had when he was first transferred. Furthermore, an increase in the use of parenteral opioid pain medications was observed despite an improvement in the patient’s functional and neurological status, raising concern for chemical coping. Defined as an excessive use of medications to manage psychological distress or suffering, chemical coping can raise significant safety concerns. Interventions targeting emotional processing have been efficacious in decreasing the effects of somatization and chemical coping and are regularly utilized by the IDT (Reddy et al. 2014; Yusufov et al. 2023). In the case of our patient, daily visits with our psychosocial support team, including a counselor and chaplain, resulted in a decreased need for intravenous opioids, a smooth transition to an oral regimen, and a significant decrease in his MEDD by the time of discharge.

Continued access to an acute hospital level of care along with a focus on symptom-oriented care allows for ancillary hospital services to continue judiciously, further bridging the gap between anticipated in-hospital death and discharge home. Our patient was able to receive services from several other disciplines while in the APSCU, including integrative medicine, physical and occupational therapies, interventional radiology, and medical oncology. The available support services are more than what hospice can routinely provide, making the APSCU a unique level of care. Prior studies have demonstrated benefit from intensive physical and occupational therapies despite poor prognosis (Fu et al. 2021). During our patient’s time in the APSCU, he was able to recover from critical illness enough to ambulate independently and transition safely home. Our team was able to have other medical specialists address time-sensitive barriers to safe discharge as well, including procedural replacement of his drainage gastrostomy tube and a visit from his medical oncologist to reaffirm his plan of care, providing peaceful acceptance of their decision to pursue comfort-oriented care.

APSCUs result in significant symptom improvement and higher rates of live discharges than anticipated (Mercadante et al. 2025). A retrospective observational study reviewing 1440 patient medical records from an APSCU at a comprehensive cancer center revealed that 69% of these patients were successfully discharged from the hospital and experienced a statistically significant improvement in symptom burden 7 days after APSCU transfer (Jung et al. 2022). Another study showed that 66% of patients transferred to the APSCU were successfully discharged (Hui et al. 2010). The sophisticated intensive symptom management through the interdisciplinary approach present in the APSCU allowed for patients like ours, who are otherwise unable to be discharged from a higher level of care, to be safely discharged home with improved quality of life.

As our case demonstrates, APSCUs provide an ideal transition unit in the hospital for the potential stabilization of a patient’s condition and ultimate discharge home. The multidisciplinary team approach to patient care allowed us to address the patient’s physical and emotional well-being: for example, we were able to identify and successfully address chemical coping and facilitate appropriate expectation-setting regarding discharge. This sophisticated care led to the patient’s successful discharge home with an excellent quality of life. This case adds to the growing body of literature demonstrating the benefits of an APSCU in the cancer population. We continue to support and advocate for APSCUs in all comprehensive cancer centers, with an ideal future expansion to all acute care hospitals.
